# The Poor Man's Home.—XXI

**Published:** 1898-01-15

**Authors:** 


### Modern Sociology.

### THE POOR MAN'S HOME.—XXI.

### Housing in Germany (*concluded*).

One has a certain hesitation in describing the workmen's houses at Mulhouse as being examples of German benevolence. Mulhouse is in Alsace, and M. Dollfus, the manufacturer, whose workmen's dwellings we propose to describe, was, when he began them, a Frenchman by nationality. Moreover, one of the contributors to the fund that helped to build them was the Emperor Napoleon III. Nevertheless, as they are now to be regarded as belonging to the Fatherland, we have included them under housing in Germany.

The "Société Mulhousienne des Cités Ouvrières" dates from 1853. In that year M. Jean Dollfus, having made a study of what had been done in the way of housing the working classes in England, came back to his native town determined to follow the example that Britain had set. By way of finding out what was the best kind of house for the people he proposed to benefit, he engaged an architect, M. Emile Muller, to erect four houses of different types, and, after a trial of several months, the tenants were consulted as to the one they preferred. The style finally adopted is now generally known as the Mulhouse type. One would not say, judging from the plan, that the chosen type was perfect; on the contrary, it fails to provide for that through ventilation which is always a desideratum, and never more so than in the homes of the poor. In a large city the Mulhouse type might be unsatisfactory, but in a small town it need not of necessity be unhealthy, and it combines in an exceptional degree the advantages, as a rule mutually exclusive, of independence and companionship. In the centre of a plot of ground a square building is erected. This is divided into four dwellings, each dwelling occupying a corner of the building. This plan gives each family two exposures, and two doors. There is much more of the feeling of owning a house than can be obtained from the occupation of a mere flat, and, of course, each angle has its own garden adjoining the house; but, on the other hand, while every room is open to the air, there is no absolutely through ventilation possible. The houses themselves are two storeys high. Each house contains four rooms, an attic, and a cellar. The rent is 187½ francs per annum (£7 10s. 9½d.), and by an additional payment of 6 francs per month (4s. 10d.), the tenant becomes the owner of the house in fifteen years. There are in Mulhouse three-room houses also, which cover more ground, but are of only one storey. The rent of these is 168 francs per annum (£6 15s. 1d.), but the additional sum for purchase price, viz., 6 francs per month for fifteen years, is the same as in the larger houses. In all these houses the water is drawn from wells outside the houses, and the privies are outside.

Mulhouse possesses houses which are not of what is known as the "Mulhouse" type, built in rows in the common style. The best of these are two storeys high, and each set of two is divided from the others by a heavy wall. These are larger than the corner houses we have spoken of, and contain a kitchen and four, or, even in some cases, five or six rooms, besides attics, which can be used as bedrooms. These houses have sculleries in a back building attached to the house, and there also are the privies placed. There is yet another type of house, which must be condemned by sanitary science. In it the houses are placed in rows, back to back. They are two storeys high, and have a large garden in front; but this cannot be considered as compensating for the badness, from a sanitary point of view, of the construction. Indeed, it is a pity when faulty houses, such as these, look attractive in any way. It may be said here, that the donation of 300,000 francs made by the Emperor Napoleon III. to the enterprise was expended in providing sewers, baths, wells, public washhouses, and the like, and the constructing of streets, the planting of trees, and the various kinds of expenditures which are usually undertaken by municipalities. The Mulhouse dwellings return a dividend of 4 per cent., and they are usually sold while still in course of erection, although at first there was considerable difficulty in selling any.

The German Government has built some small but good dwellings for its employés in the Marine Service. A peculiarity of them is that a fair proportion are intentionally built larger than the family that is to occupy them will need, in order that lodgers may be taken. In a service where a large proportion of those employed are certain to be single, this is a wise arrangement, as these single men might have some difficulty in finding the accommodation they require in the neighbourhood. Another advantage of being a Government servant is that the water used in these houses, which are situated at Friedrichsort, is periodically examined by a bacteriologist. This sanitary precaution must, however, be counterbalanced by the sanitary defect of the privies being destitute of flushing arrangements. There is not even a pit, as in most German dwellings, but only a barrel which is emptied weekly. In the houses intended for one family only there are, as a rule, three apartments—a kitchen, a living-room, and a bedroom. The houses are built in two storeys, but within the front door are two inner ones; that on one side leading into the house on the lower floor, while the other opens on to a stair, which conducts to the upper. As a small hall is thus cut off the lower house, the kitchen there is smaller than upstairs. It measures 8 ft. 10 in. by 7 ft. 10 in., while that on the upper floor is the same width, but is 13 ft. 5 in. long. The other rooms are the same size on each flat, viz., the living room 13 ft. 5 in., and the bedroom 13 ft. 3 in. in length, while each is 11 ft. 10 in. in width. The rent of a house of this size is 144 marks (£7 2s. 9½d.) per annum. The dwellings in which lodgers may be kept are of five rooms, and in each of the rooms which are meant to be let there are stoves provided on which food may be kept warm. In all the houses cooking-ranges are provided, and tiled stoves in the living rooms. Among other advantages which the occupants possess are the use of laundries, provided in the proportion of one to every two families, a public kitchen, a play-ground, a park, and a sea bathing establishment. About 12 per cent. of the earnings of the occupants go in rent. This is at least 3 per cent. less than is usual elsewhere, and as we have seen, there are many places where the proportion of money paid for rent is very much larger.

